# Comparison of apoptotic responses in *Blastocystis* sp. upon treatment with Tongkat Ali and Metronidazole

**DOI:** 10.1038/s41598-021-81418-x

**Published:** 2021-04-09

**Authors:** Sonal Girish, Suresh Kumar, Norhaniza Aminudin, Najihah Mohd Hashim

**Affiliations:** 1 Department of Parasitology, Faculty of Medicine, Universiti Malaya, 50603 Kuala Lumpur, Malaysia; 2 Faculty of Science and Universiti Malaya Centre for Proteomics Research, Medical Biotechnology Laboratory, Faculty of Medicine, Institute of Biological Sciences, Universiti Malaya, 50603 Kuala Lumpur, Malaysia; 3 Department of Pharmaceutical Chemistry, Faculty of Pharmacy, Universiti Malaya, Kuala Lumpur, 50603 Malaysia; 4 Centre for Natural Products and Drug Discovery (CENAR), Universiti Malaya, Kuala Lumpur, 50603 Malaysia

**Keywords:** Drug discovery, Microbiology, Medical research

## Abstract

*Blastocystis* sp. infection, although many remain asymptomatic, there is growing data in recent studies that suggests it is a frequent cause of gastrointestinal symptoms in children and adults. This proposes that treatment against this infection is necessary however metronidazole (MTZ), which is the current choice of treatment, has expressed non-uniformity in its efficacy in combating this infection which has led to the study of alternative treatment. In our previous study, it was established that Tongkat Ali fractions exhibited promising anti-protozoal properties which leads to the current aim of the study, to further narrow down the purification process in order to identify the specific active compound promoting the anti-protozoal effect through HPLC analysis. Based on the data analysis and in-vitro susceptibility assay, the collected Tongkat Ali fraction that demonstrated anti-*blastocystis* property was shown to contain eurycomanone. Previous studies have suggested that there is a mechanism in *Blastocystis* sp. that regulates the apoptotic process to produce higher number of viable cells when treated. In reference to this, our current study also aims to investigate the apoptotic response of Tongkat Ali extract and eurycomanone across different subtype groups with comparison to MTZ. Based on our investigation, both Tongkat Ali extract and eurycomanone induced the high apoptotic rate however exhibited a reduction in viable cell count (*p* < 0.05) when compared to MTZ. This study suggests that there is potential in developing a standardized treatment regardless of subtype variations which makes Tongkat Ali extract a promising anti-protozoal treatment against all *Blastocystis* sp. subtype groups.

## Introduction

Programmed Cell Death (PCD) is an important biochemical process of cell elimination in which the body goes through a series of molecular steps that leads to elimination of damaged, injured or destructive cells which in turn plays a significant role for the purpose of continuous cell renewal^1,2^. Apoptosis, a form of PCD, has previously been described as an orchestrated collapse of a cell characterised by membrane disintegration, cell shrinkage, chromatin condensation, nuclear fragmentation, chromosomal DNA fragmentation which is then followed by rapid engulfment of the corpse by neighbouring cells^3–5^. While metazoan apoptosis is widely studied and reported, there is increasing data on the apoptosis markers found in unicellular organisms such as *Blastocystis* sp.^6^, *Chlammydomonas*^7^, *Dictyostelium*^8^, *Giardia*^6^, *Leishmania*^9,10^, *Plasmodium*^11,12^, and *Trypanosoma*^13^. Protozoan programmed cell death or apoptosis is vital to ensure the continuity of the parasite life cycle and its pathogenicity. Apoptosis in protozoan parasites is well characterized by a unique design of morphological alterations in cytoplasm and nucleus and several of these features have been described in *Blastocystis* sp. exposed to cytotoxic drug such as metronidazole (MTZ)^14,15^.

A study by Raman et al.^16^ revealed that an increase in the number of mithochondrion like organelle (MLO) in symptomatic *Blastocystis* sp*.* subtype (ST3) isolates were observed when treated with MTZ. The presence of MLOs’ implicated that these treated parasites need greater energy for the purposes of internal reorganization in response to the drug. In another study by Dhurga et al.^17^, a variation in apoptotic response was observed when 4 different ST’s of *Blastocystis* sp. were investigated for rate of apoptosis when treated with MTZ. Amongst the four, ST3 presented with the highest significant increase in the number of *Blastocystis* sp. cells upon treatment with MTZ which coincided with a previous study^16^ postulating that treatment with MTZ can aggregate the growth of *Blastocystis* sp. cells increasing its pathogenic potential. This suggests that there is a survival mechanism regulated by *Blastocystis* sp. cells in response to treatment hence producing higher number of viable cells when treated with MTZ.

In our previous study^18^, amongst the many Malaysian popular herbal extract that were screened for its anti-*blastocystis* effect, significant growth inhibition was observed upon treatment with Tongkat Ali (*Eurycoma longifolia*) with results comparable to a reference drug. Besides being a natural aphrodisiac, Tongkat Ali has many other medicinal benefits mainly due to its vast composition of secondary metabolites^19,20^. Extensive studies have reported the therapeutic effects that Tongkat Ali possesses such as anti‐malarial, anti‐tumor, anti-bacterial properties^21,22^. Our study was also the first to demonstrate the anti-protozoal property of Tongkat Ali against *Blastocystis* sp. Bringing it a step ahead, in this current study we attempt to further isolate the compounds present in Tongkat Ali crude extract in order to identify the active compound of the plant extract specifically responsible for the anti-protozoal activity against *Blastocystis* sp. As mentioned earlier, treatment with MTZ against *Blastocystis* sp*.* is postulated to be able to activate the apoptotic mechanism where in turn increases the proliferation of viable cells. In reference to this, the current study also aims to investigate and compare the apoptotic response of the anti-protozoal promoting compound found in Tongkat Ali extract against different groups of *Blastocytsis* sp. subtypes (ST) with MTZ as the reference drug.

## Materials and methods

### Parasite culture and subtyping

A total of nine human-derived *Blastocystis* sp. isolates comprising of 3 different ST groups (ST1, ST2 and ST3) were obtained and isolated from individuals. Parasites were maintained through in vitro cultivation in Jones’ medium supplemented with 10% horse serum and incubated at 37 °C^23^. Parasites were sub-cultured once every 3–4 days for at least 1 month prior to this study. DNA was extracted directly from the culture samples using the QIAGEN Stool Mini Kit (QIAGEN, Hilden, Germany). PCR reaction was performed using the seven pairs of sequenced-tagged site (STS) primers (SB83, SB155, SB227, SB332, SB340, SB336 and SB337). Only isolate of a single subtype is included in this study^18^.

### HPLC analysis

HPLC (Agilent 1260 Infinity) with diode array detector (DAD) was utilized with ZORBAX Eclipse XBD-C18 column (Analytical 4.6 × 250 mm, 5 um). With reference and slight modification to the protocol previously described^22^, briefly, the column temperature was maintained at 30 °C with an injection volume of 10 µl. The mobile phase consisted of isocratic mixture of water (0.1%TFA) and acetonitrile (86:14) with a flow rate of 0.8 ml/min. The UV detector was operated at a wavelength 254 nm. The HPLC was performed on a LC-20 Prominence system (Shimadzu, Kyoto, Japan) with a SPD-20A UV–VIS detector using LC-Solution software.

### ^1^H-NMR analysis

The sample preparation for 1H-NMR analysis was according to the manufacturer’s protocol. ^1^H-NMR measurements were performed with a 300 MHz NMR spectrometer at 298 K using a PULprog Zg30 on a Bruker Fourier 300, with MeOD as the solvent of choice. The chemical shifts were recorded in ppm (d units) in relation to TMS as an internal standard. Pulse conditions were as follows for ^1^H-NMR: dwell time (DW) 81.920 µs, acquisition time (AQ) 5.3687091 s, number of transients (NS) 16, recycle delay (RD) 2.000s^24^.

### Induction of cell death by different treatment (MTZ, TA, TA fraction)

*Blastocystis* sp. isolates were subjected to MTZ (2-methyl-5nitroimidazole-1-ethanol), *Eurycoma longifolia* (TA) and TA fraction treatment. Stock solutions of these treatments were prepared in distilled water and were further diluted to obtain the desired concentrations. Then, 1 × 10^5^ *Blastocystis* sp. cells/ml were introduced into a 1.5 ml microcentrifuge tube (Axygen Biosciences, Union City, CA, USA) containing a final concentration of 0.01 mg/ml and 0.1 mg/ml TA and TA fraction and 0.001 mg/ml and 0.01 mg/ml MTZ. Cells were then harvested at 24, 48, 72, and 96 h for epifluorescence microscopy analysis^17^.

### Detection of apoptotic, late apoptotic stage and necrotic cells

Detection of apoptotic, late apoptotic stage and necrotic cells was done using an Apoptosis, Necrotic and Healthy Cells Qualification Kit (Biotium Inc., Hayward, CA, USA). Harvested cells were washed twice with 1 ml of PBS (pH 7.4). Then, 1 × binding buffer, ethidium homodimer III (EtD-III) (200 M in PBS), fluorescein isothiocyanate (FITC)—annexin V [250 l in Tris-EDTA buffer containing 0.1% bovine serum albumin and 0.1% NaN3 (pH 7.5)] and Hoechst 33342 (500 g/ml in PBS) were added sequentially. Samples were then observed under an Olympus BX 51 epifluorescence microscope (Olympus, Wetzlar, Germany) using image analyser software. Results of cells undergoing apoptosis, late apoptosis stage and necrosis were quantified with regard to percentage of apoptotic, late apoptotic stage and necrotic cells per 100 cells. Hoechst 33342, which is a cell-membrane permeant, minor-groove-binding DNA stain, was used as a substitute for the nucleic acid stain DAPI (4, 6-diamidino2-phenylindole). It stains the nuclei of both apoptotic and necrotic cells. Apoptotic cells were determined by FITC–annexin V staining, which binds to exposed phosphotidylserine (PS) in a cell undergoing apoptosis. This dye is used together with EtD-III, a superior alternative to propidium iodide (PI). Absence of PI staining signals that the membrane integrity is not compromised. Healthy cells are stained blue by Hoechst 33342 stain only. Apoptotic cells are stained with both blue (Hoechst 33342) and green (FITC–annexin V). Cells stained blue, green and red (EtD-III) are late apoptotic stage cells while cells stained blue and red are necrotic cells^17^.

### Statistical analysis

Statistical analysis was carried out using SPSS Statistics 20.0 software (SPSS Inc., Chicago, IL, USA). Independent Student’s t-test was used where *p* value < 0.05 was considered statistically significant^18^.

## Results

### Identifying the anti-protozoal promoting compound against *Blastocystis* sp. in TA extract

To further identify and quantify the anti-protozoal promoting compound against *Blastocystis* sp., TA extract was subjected to reverse phase HPLC to separate and collect the fractions. Following the separation of TA extract fraction by RP-HPLC, eluted peaks were collected and tested for the highest efficacy of anti-protozoal promoting active compound against *Blastocystis* sp.

**Figure 1:**
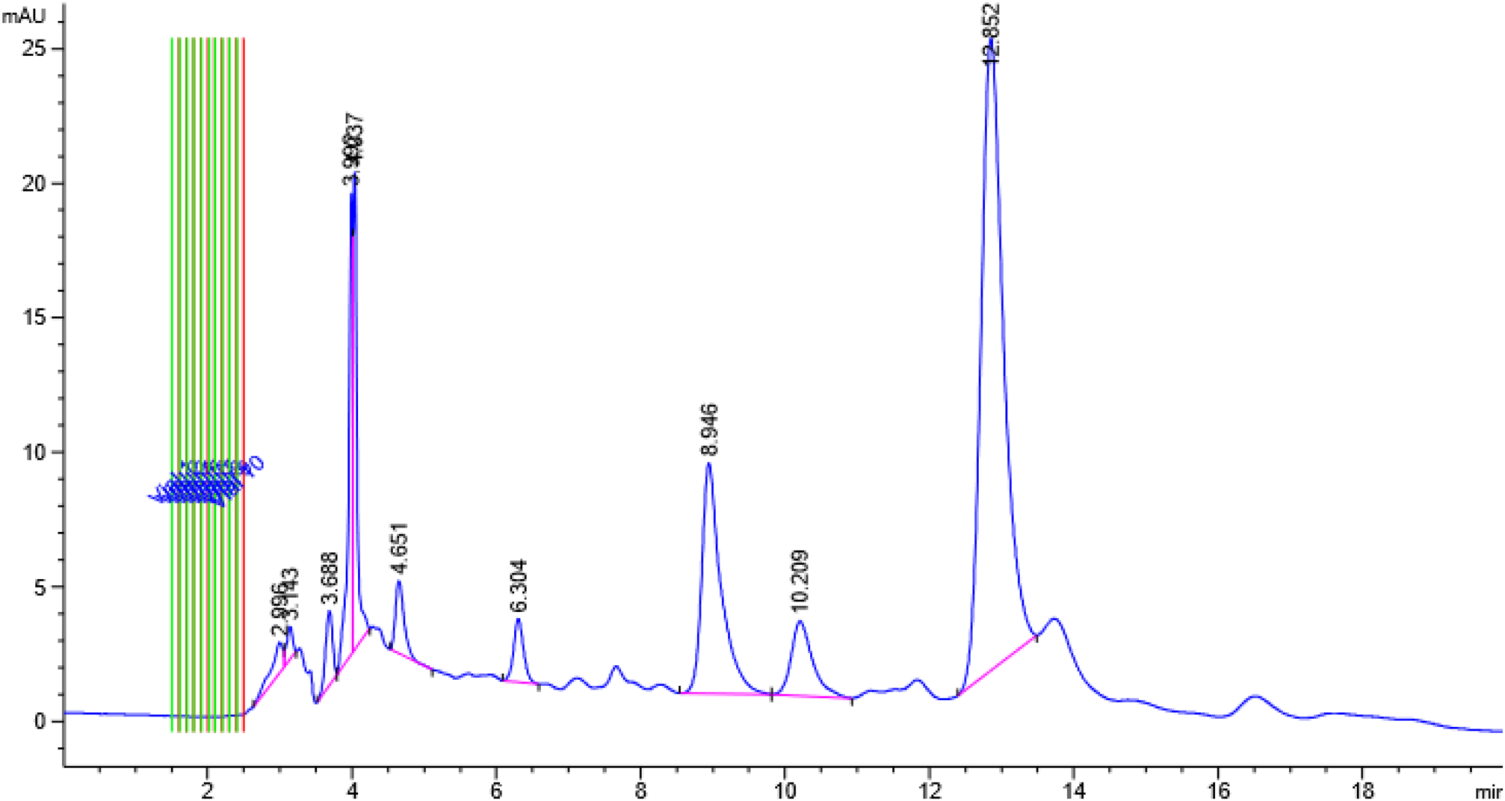
HPLC chromatogram of TA extract.

**Figure 2:**
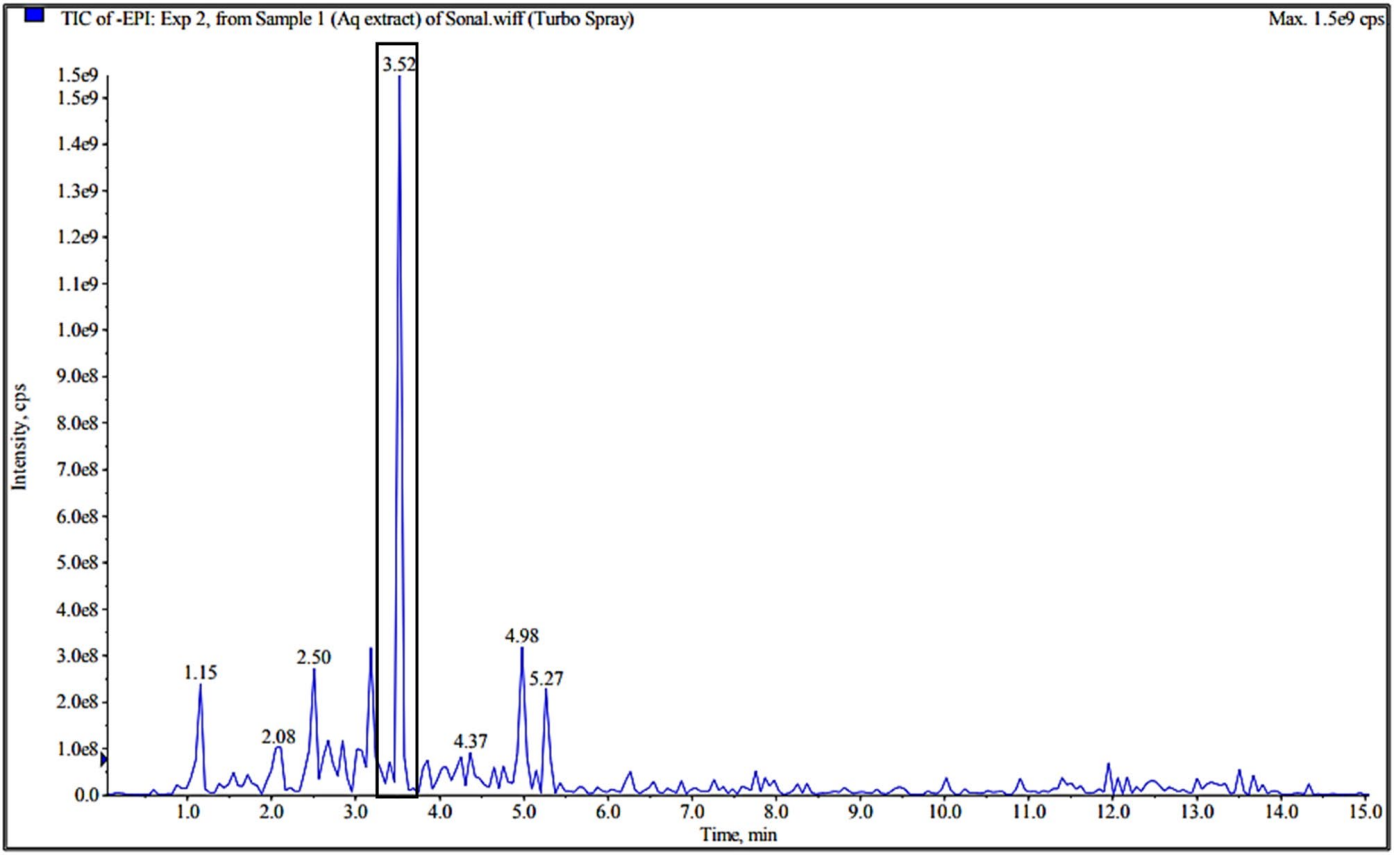
Total Ion Chromatogram (TIC) LC–MS analysis of compounds identified in TA extract.

**Figure 3:**
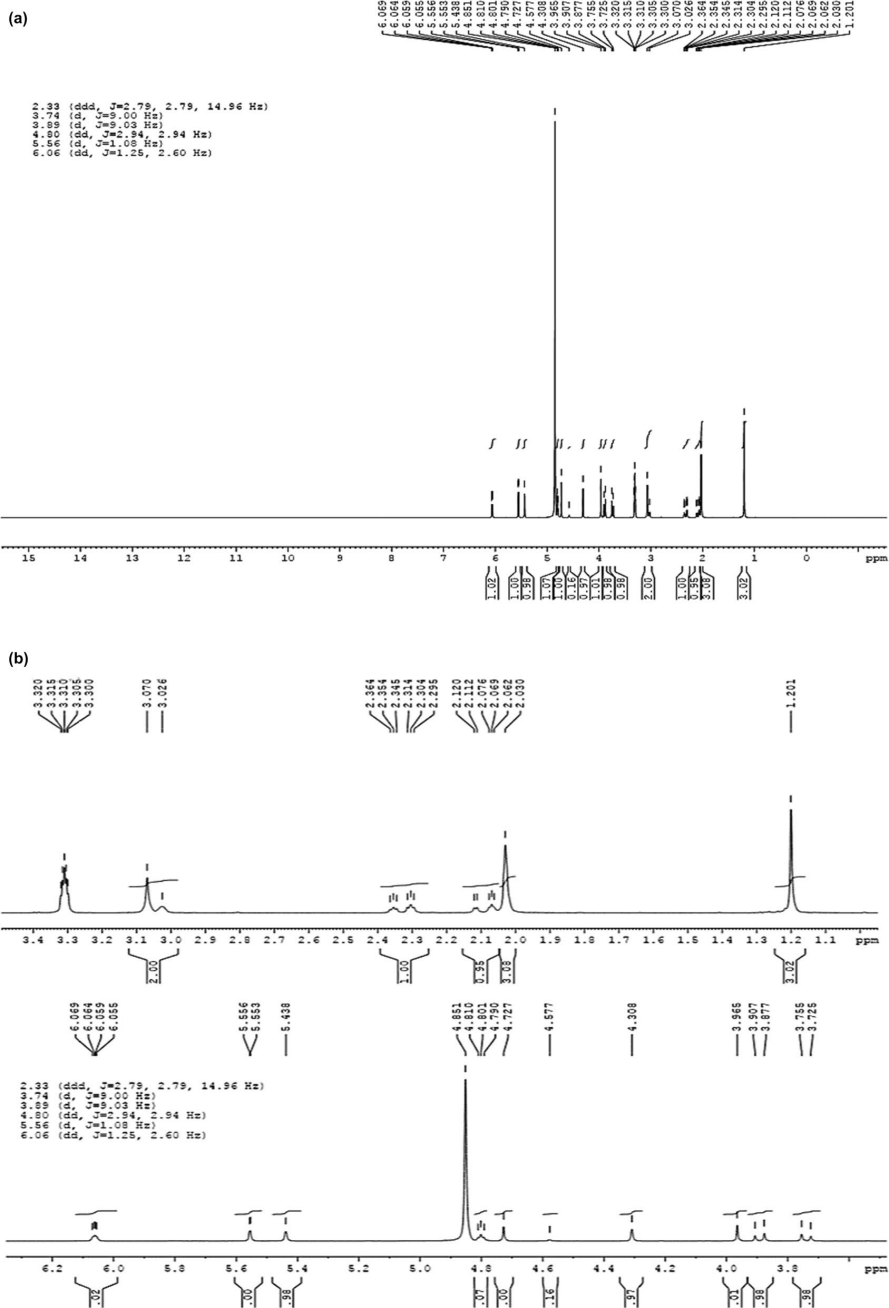
(**a**,**b**) ^1^H-NMR spectrum of eurycomanone in MeOD.

Referring to Fig. 1, from all the eluted peaks, the fraction that showed the highest anti-protozoal activity against *Blastocystis* sp. ST 1, 2 and 3 in in-vitro study was showed to contain eurycomanone (EU). Figure 2 shows the LC–MS analysis of TA extract and the peak of interest that was identified as EU with reference to the standard chromatogram developed by Nhan and Loc^22^. This was further verified through the ^1^H-NMR analysis as presented in Table 1. The spectral data based on Fig. 3a,b was cross referenced based on a previously published paper^24^.

**Table 1:** Percentage of viable, apoptotic, late apoptotic stage and necrotic *Blastocystis* sp. cells after 72 h of culture.

Cell stage	Viable						
Subtype	Untreated	MTZ 0.001	MTZ 0.01	TA 0.01	TA 0.1	EU 0.01	EU 0.1
1	35.67 ± 1.15	33.33 ± 4.16	22.67 ± 2.08	23.00 ± 1.73	17.67 ± 0.58	19.33 ± 0.58	12.33 ± 0.58
2	29.67 ± 0.57	39.33 ± 2.88	25.33 ± 2.52	21.67 ± 1.53	18.33 ± 1.16	22.00 ± 1.0	13.33 ± 1.53
3	25.67 ± 5.03	29.00 ± 1.00	23.00 ± 1.73	22.00 ± 2.65	15.00 ± 1.00	17.67 ± 0.58	13.67 ± 1.16

Cell stage	Apoptotic						
Subtype	Untreated	MTZ 0.001	MTZ 0.01	TA 0.01	TA 0.1	EU 0.01	EU 0.1
1	39.33 ± 1.16	47.00 ± 4.36	49.00 ± 2.65	51.00 ± 0.00	58.00 ± 0.00	51.00 ± 0.00	66.00 ± 0.00
2	43.33 ± 2.31	41.33 ± 1.16	50.00 ± 2.00	54.33 ± 1.16	58.00 ± 0.00	51.67 ± 2.31	66.00 ± 0.00
3	41.67 ± 2.06	45.33 ± 0.58	58.00 ± 0.00	53.67 ± 2.31	61.00 ± 1.00	56.67 ± 2.08	66.00 ± 1.73

Cell stage	Late apoptotic						
Subtype	Untreated	MTZ 0.001	MTZ 0.01	TA 0.01	TA 0.1	EU 0.01	EU 0.1
1	9.00 ± 1.73	10.33 ± 0.58	17.00 ± 1.73	16.00 ± 0.00	13.33 ± 1.16	14.00 ± 0.00	10.00 ± 0.00
2	15.67 ± 0.58	14.33 ± 3.06	14.00 ± 0.00	13.67 ± 1.16	13.33 ± 1.16	15.67 ± 0.58	11.00 ± 1.00
3	19.67 ± 4.04	13.00 ± 1.00	12.67 ± 3.79	14.67 ± 0.58	13.67 ± 0.58	15.00 ± 1.73	10.33 ± 0.58

Cell stage	Necrotic						
Subtype	Untreated	MTZ 0.001	MTZ 0.01	TA 0.01	TA 0.1	EU 0.01	EU 0.1
1	16.00 ± 1.73	9.33 ± 0.58	11.33 ± 1.16	10.00 ± 1.73	11.00 ± 1.00	15.67 ± 0.58	11.67 ± 0.58
2	11.33 ± 1.56	5.00 ± 2.65	10.67 ± 2.08	10.33 ± 0.58	10.33 ± 1.16	10.67 ± 2.08	9.67 ± 1.16
3	13.00 ± 0.00	12.67 ± 0.58	9.00 ± 1.00	9.67 ± 0.58	10.33 ± 0.58	10.67 ± 1.16	10.00 ± 0.00

Data are expressed as the mean ± SD.

**Figure 4:**
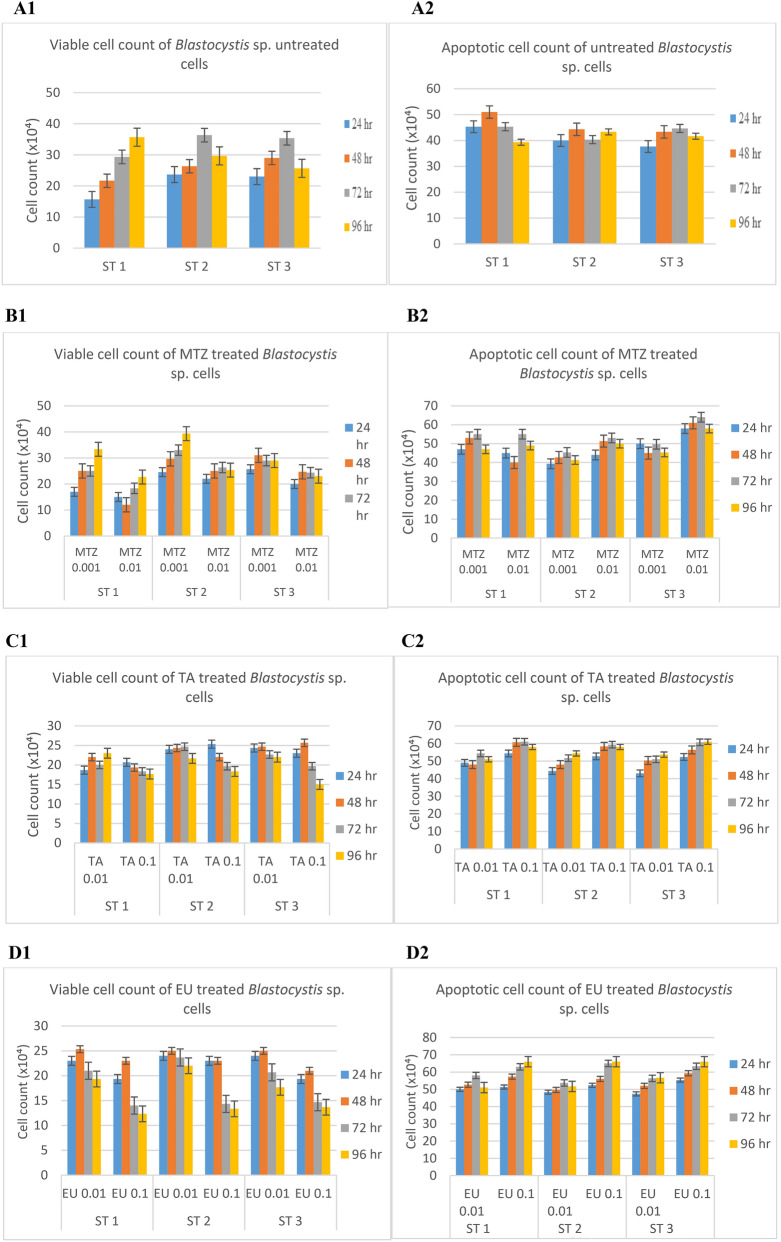
Number of viable cells and rate of apoptosis in *Blastocystis* sp.: (**A1**,**A2**) untreated cells; (**B1**,**B2**) cells treated with 0.01 mg/ml and 0.001 mg/ml MTZ; (**C1**,**C2**) cells treated with 0.1 mg/ml and 0.01 mg/ml TA; (**D1**,**D2**) cells treated with 0.1 mg/ml and 0.01 mg/ml EU.

Based on results observed in Fig. 4 (A1–D2), all three treatments induced apoptosis in all subtypes of *Blastocystis* sp. isolates. The rate of apoptosis across all three subtypes correlated with the increase in concentration of the treatments as the higher concentrations produced higher number of apoptotic cells.

Referring to Fig. 4, the pattern of viable and apoptotic cell count of MTZ treated cells varied in comparison to TA and EU treated cells. When treated with MTZ, the viable cell count of treated cells increased as apoptotic cell count increased. In contrary, treatment of cells with TA and EU saw a reduction in viable cell count (*p* < 0.05) as apoptotic cells elevated. In MTZ treated (0.01 mg/ml) cells, an increase of 53%, 14% and 15% of viable cell count was observed in ST 1, 2 and 3 respectively from 24 to 96 h. In TA treated (0.1 mg/ml) cells, the viable cell count reduction of 14.2%, 28%, and 34.8% was observed in ST 1, 2 and 3 respectively after 96 h of culture. In EU treated (0.1 mg/ml) cells a higher percentage of viable cell count reduction was observed with 37%, 44% and 32% in ST 1, 2 and 3 respectively.

Referring to Table 2, the figures show that the apoptotic rate was significantly (*p* < 0.05) higher in EU treated cells when compared to TA and MTZ across all 3 subtypes as well as lowest cell viability rate which deduces that apoptosis induced by EU triggers the inhibition of cell growth. Treatment with EU also saw uniformity in terms of viability rate and apoptotic rate across different subtypes.

**Table 2:** Percentage of viable, apoptotic, late apoptotic stage and necrotic *Blastocystis* sp. cells after 72 h of culture.

Cell stage	Viable						
Subtype	Untreated	MTZ 0.001	MTZ 0.01	TA 0.01	TA 0.1	EU 0.01	EU 0.1
1	35.67 ± 1.15	33.33 ± 4.16	22.67 ± 2.08	23.00 ± 1.73	17.67 ± 0.58	19.33 ± 0.58	12.33 ± 0.58
2	29.67 ± 0.57	39.33 ± 2.88	25.33 ± 2.52	21.67 ± 1.53	18.33 ± 1.16	22.00 ± 1.0	13.33 ± 1.53
3	25.67 ± 5.03	29.00 ± 1.00	23.00 ± 1.73	22.00 ± 2.65	15.00 ± 1.00	17.67 ± 0.58	13.67 ± 1.16

Cell stage	Apoptotic						
Subtype	Untreated	MTZ 0.001	MTZ 0.01	TA 0.01	TA 0.1	EU 0.01	EU 0.1
1	39.33 ± 1.16	47.00 ± 4.36	49.00 ± 2.65	51.00 ± 0.00	58.00 ± 0.00	51.00 ± 0.00	66.00 ± 0.00
2	43.33 ± 2.31	41.33 ± 1.16	50.00 ± 2.00	54.33 ± 1.16	58.00 ± 0.00	51.67 ± 2.31	66.00 ± 0.00
3	41.67 ± 2.06	45.33 ± 0.58	58.00 ± 0.00	53.67 ± 2.31	61.00 ± 1.00	56.67 ± 2.08	66.00 ± 1.73

Cell stage	Late apoptotic						
Subtype	Untreated	MTZ 0.001	MTZ 0.01	TA 0.01	TA 0.1	EU 0.01	EU 0.1
1	9.00 ± 1.73	10.33 ± 0.58	17.00 ± 1.73	16.00 ± 0.00	13.33 ± 1.16	14.00 ± 0.00	10.00 ± 0.00
2	15.67 ± 0.58	14.33 ± 3.06	14.00 ± 0.00	13.67 ± 1.16	13.33 ± 1.16	15.67 ± 0.58	11.00 ± 1.00
3	19.67 ± 4.04	13.00 ± 1.00	12.67 ± 3.79	14.67 ± 0.58	13.67 ± 0.58	15.00 ± 1.73	10.33 ± 0.58

Cell stage	Necrotic						
Subtype	Untreated	MTZ 0.001	MTZ 0.01	TA 0.01	TA 0.1	EU 0.01	EU 0.1
1	16.00 ± 1.73	9.33 ± 0.58	11.33 ± 1.16	10.00 ± 1.73	11.00 ± 1.00	15.67 ± 0.58	11.67 ± 0.58
2	11.33 ± 1.56	5.00 ± 2.65	10.67 ± 2.08	10.33 ± 0.58	10.33 ± 1.16	10.67 ± 2.08	9.67 ± 1.16
3	13.00 ± 0.00	12.67 ± 0.58	9.00 ± 1.00	9.67 ± 0.58	10.33 ± 0.58	10.67 ± 1.16	10.00 ± 0.00

Data are expressed as the mean ± SD.a

## Discussion

In the present study, the fraction collected through HPLC analysis that was found to exhibit the highest anti-protozoal activity against *Blastocystis* sp. isolates when tested in-vitro showed to contain eurycomanone. This current study is the first to identify the anti-protozoal property of eurycomanone against *Blastocystis* sp. isolates and the results were comparable to that of MTZ. Eurycomanone, a phytochemical that is exclusively found in Tongkat Ali extracts is reported to be the most abundant quassinoid in the roots of Tongkat Ali^25–29^. The therapeutic properties of *Tongkat Ali* that have previously been reported, such as male fertility enhancement^26^, antimalarial^30,31^, cytotoxic^32^, antiproliferative^33,34^ and antiulcer^35^ effects, are largely attributed to the quassinoids group, specifically eurycomanone. In a study conducted by Chan and co-workers^36^, four quassinoids including eurycomanone, 13,21-dihydroeurycomanone, 13αepoxyeurycomanone, eurycomalactone, and an alkaloid, 9- methoxycanthin-6-one from the root extracts of Tongkat Ali were isolated in order to determine its antiprotozoal efficacy. The antiprotozoal activity of these compounds was tested against chloroquine-resistant Gombak A isolate of *Plasmodium falciparum*. Amongst the compounds tested, eurycomanone, isolated from the root extract of TA, exhibited outstanding anti-plasmodial activity.

**Figure 5:**
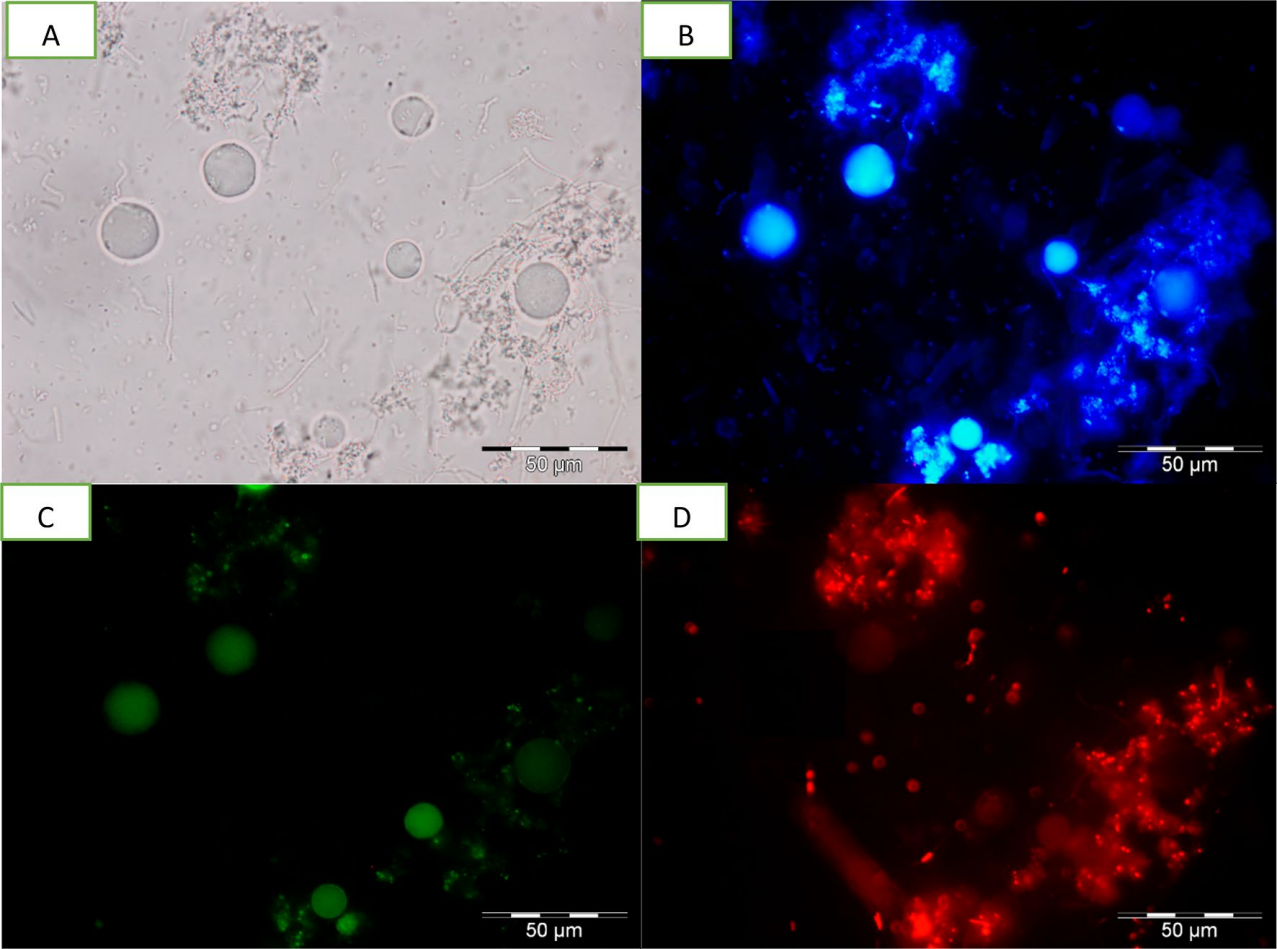
Images of untreated *Blastocystis* sp. undergoing apoptosis viewed under epifluorescent microscope; (**A**) Bright field; (**B**) stained using Hoechst stain; (**C**) stained using FITC Annexin (V); (**D**) stained using Propidium Iodide.

**Figure 6:**
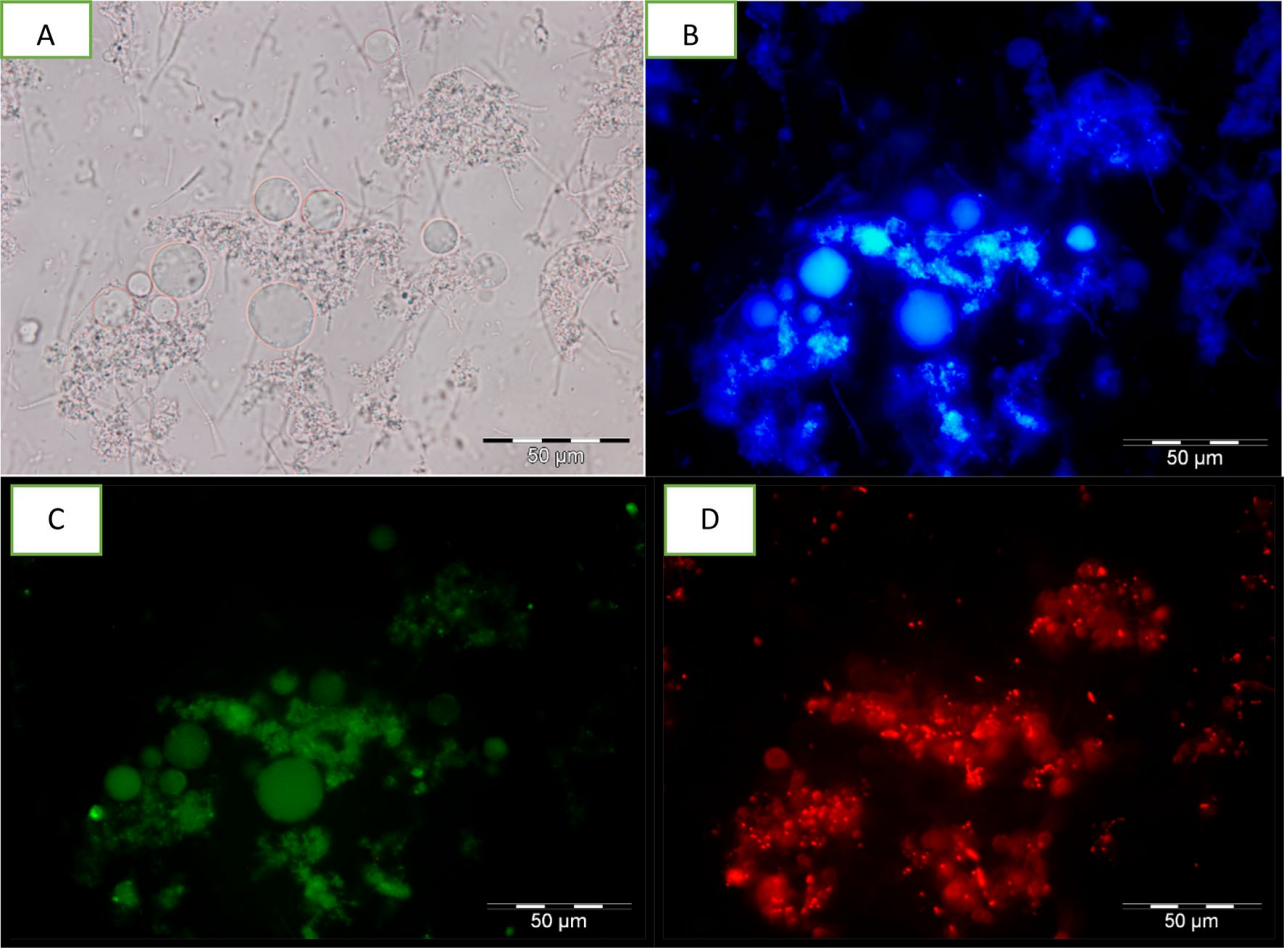
Images of MTZ treated (0.01 mg/ml) *Blastocystis* sp. undergoing apoptosis viewed under epifluorescent microscope; (**A**) Bright field; (**B**) stained using Hoechst stain; (**C**) [stained using FITC Annexin (V); (**D**) stained using Propidium Iodide.

**Figure 7:**
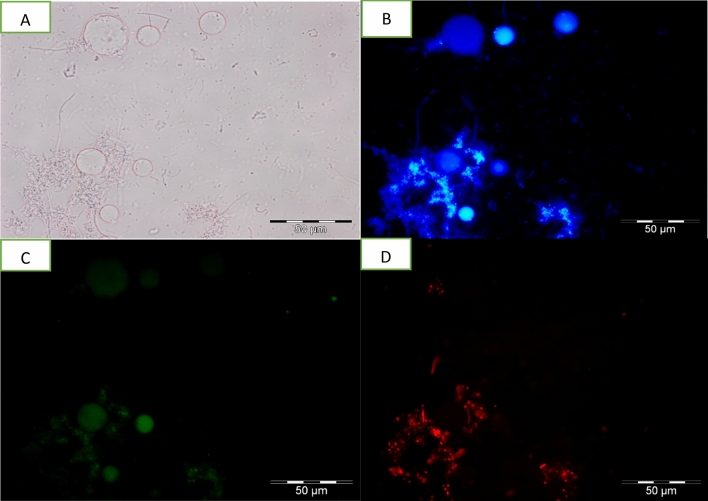
Images of TA treated (0.01 mg/ml) *Blastocystis* sp. undergoing apoptosis viewed under epifluorescent microscope; (**A**) Bright field; (**B**) stained using Hoechst stain; (**C**) [stained using FITC Annexin (V); (**D**) stained using Propidium Iodide.

**Figure 8:**
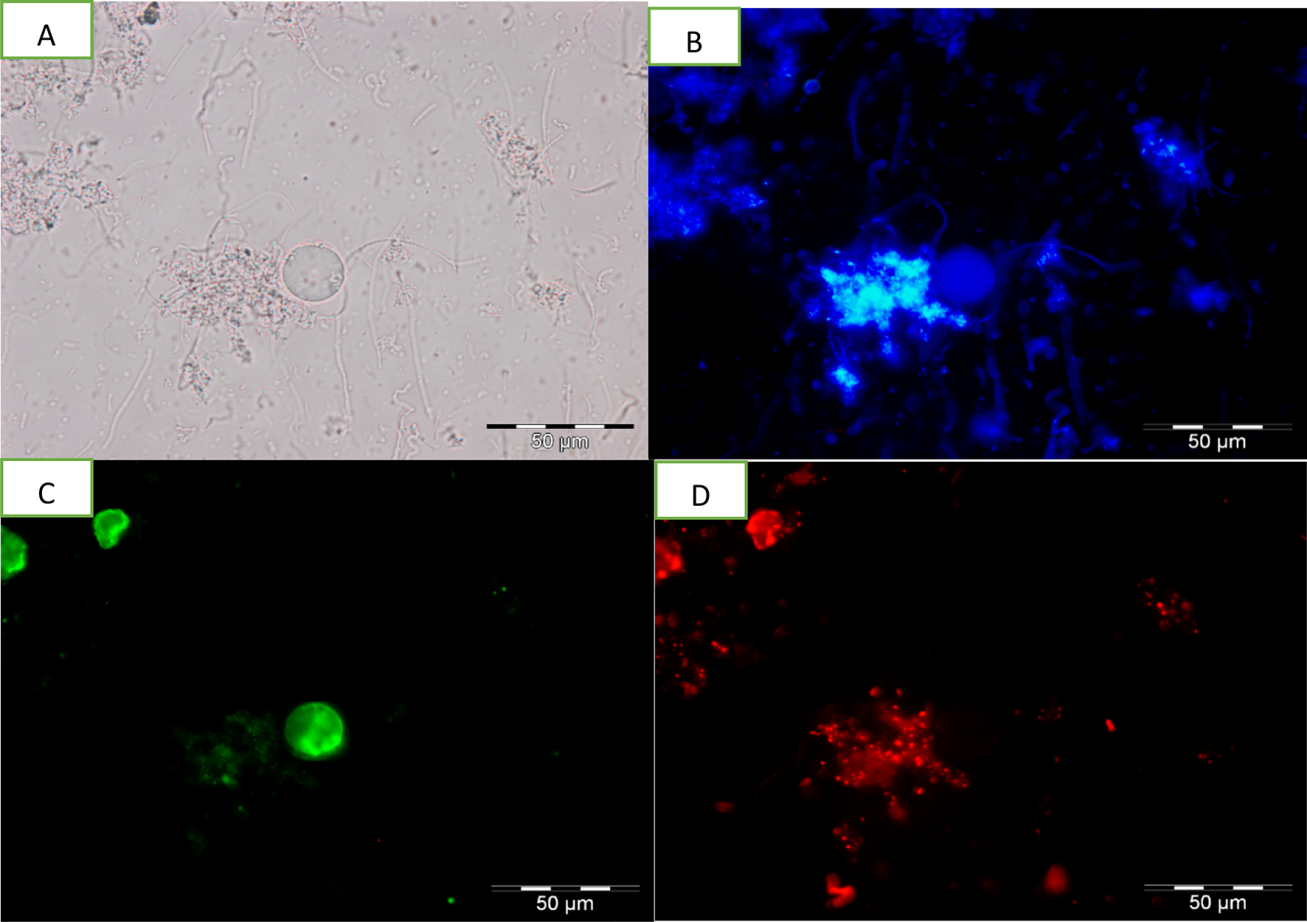
Images of EU treated (0.01 mg/ml) *Blastocystis* sp. undergoing apoptosis viewed under epifluorescent microscope; (**A**) Bright field; (**B**) stained using Hoechst stain; (**C**) stained using FITC Annexin (V); (**D**) stained using Propidium Iodide.

Apoptotic studies are an essential part of any drug development as it is an important contribution that influences the chemotherapeutic approaches to the development of treatments. Similarly, the study on apoptotic responses of the emerging *Blastocystis* sp. infection is necessary as it provides us with clues on host-parasite interactions and pathogenesis which can further aid in the development of suitable and effective treatment. There are several analytical methods, many of which rely on fluorescence processes that have been developed to study apoptosis. In this study, referring to Figs. 5, 6, 7 and 8, the morphological changes of apoptotic cells, chromatin condensation and formation of apoptotic bodies was determined using FITC-annexin V staining. It stains apoptotic cells green by binding to PS exposed on the cell surface. Untreated cells stain homogenously with Hoecsht 33342, no fluorescence detected indicating the cells are healthy while the treated cells showed of intense fluorescence indicating that cells were apoptotic. Based on data collected, high apoptosis rate was noticed in response to all treatments, however, the correlation between viable cells and apoptotic cells varied between treatments. When treated with MTZ, the viable cell count increased with increasing rate of apoptosis, conversely treatment with TA and EU exhibited a reduction in viable cell count when rate of apoptosis increased. Nasirudeen et al.^14^, explained that MTZ induces apoptosis in *Blastocystis* sp. as a defensive mechanism used by unicellular organisms for the preservation of cell populations to ensure that some of the cells survive to propagate the genome.

The survival of these *Blastocystis* sp. cells could be a result of an efficient and effective apoptotic response to MTZ particularly through the formation of granular forms that later releases new progeny of vacuolar forms contained in their vacuoles. This suggests that granular formation during apoptosis is a self-regulatory mechanism to produce higher number of viable cells in response to treatment with MTZ^37^. Comparing the treatment responses to MTZ, in the present study, TA and EU treatments both exhibited apoptotic reaction while inhibiting the growth of *Blastocystis* sp. cells. EU has been extensively explored for its anti-cancer properties. It is one of the novel compounds with promising potencies to be developed as a new chemotherapeutic agent. This is based on the fact that chemotherapeutic agents induce cancer cell death through the mechanism of apoptosis which is one the main characteristic of EU. Alterations of intracellular mechanisms via the expressions of pro and anti-apoptotic proteins, Bax and Bcl-2 respectively, are an important hallmark in the investigation of apoptotic responses. In a study investigating the apoptotic responses of EU against HepG2 cells^38^, the effect of EU on the expression of Bcl-2 protein in HepG2 cells showed that the compound can inhibit Bcl-2 activity, as the time of exposure increases, the expression of Bcl-2 protein is lowered. Bcl-2, an anti-apoptotic gene inhibits apoptosis by barring the opening of permeability transition (PT) channels on the mitochondrion, a common pathway to necrosis and apoptosis, hence the lowering of mitochondrial potential. As a conclusion from their finding the level of Bax indirectly correlated to the decrease of Bcl-2 protein, suggesting that EU induced apoptosis through up-regulation of Bax and down-regulation of Bcl-2 level^39,40^.

Taking off with the same ideology, in the context of *Blastocystis* sp., a study conducted by Dhurga et al.^17^ showed that treatment with MTZ caused a surge in Bcl-2-like activity. It was observed at the 72 h mark in all the subtypes with subtype 3 recording the highest rate of Bcl-2-like activity. These results hypothesizes, that though TA and EU just like MTZ activates the apoptotic reactions in *Blastocystis* sp., the ability however of TA and EU to inhibit the proliferation of *Blastocystis* sp. is due to the down regulation of Bcl-2, which is a cell survival protein best known for its roles in inhibiting apoptosis thus making it a promising anti-protozoal agent against *Blastocystis* sp. infection. Besides Bax and Bcl-2 protein, another pivotal protein, the mitogen-activated protein kinase (MAPKs), plays an important role in immune responses by triggering cellular responses and production of cytokines during pathogenic infections^41^. It is postulated that infection with *Blastocystis* sp. may contribute to the pathogenesis of inflammatory intestinal diseases through the activation of inflammatory pathways in host immune cells. A study conducted by Lim et al.^42^, demonstrated for the first time that *Blastocystis* sp*.* activates MAPK pathways and induces pro-inflammatory cytokines, including IL-1β, IL-6, and TNF-α, in a subtype-dependent manner. A continuous activation of MAPK pathway can lead to excessive production of MAPK-regulated genes, uncontrolled proliferation, and unscheduled cell death. This phenomenon can be counteracted by presence of EU as treatment with EU containing extract has previously been reported to prevent the initiation of MAPK by TNFα^33^.

## Conclusion

Based on the outcome of this study, we suggest that both Tongkat Ali and eurycomanone can inhibit the growth of *Blastocystis* sp. cells and the mechanism of cell death induction was via apoptosis. This study questions many postulations on the exact mechanism of which Tongkat Ali and its compound exerts its anti-protozoal activity. In comparison to MTZ, in this study treatment with TA extract and its fraction also saw uniformity in terms of viability rate and apoptotic rate across different subtypes. This current data coincides with previous findings where a subtype dependent apoptotic rate was observed when treated with MTZ. This implies that treatment with MTZ prerequisites the need to diagnose the subtype of *Blastocystis* sp. in order for any chance of cure. However, treatment with both TA and EU eliminates this process which suggests that there is potential in developing a standardized treatment regardless of subtype variations which makes it a promising anti-protozoal treatment against all *Blastocystis* sp. subtype groups. The complexity of apoptosis induction and execution in parasites, particularly *Blastocystis* sp. still remains unsolved therefore it is necessary to further study the underlying molecular mechanism in order to extrapolate and develop an efficient anti-protozoal agent against *Blastocystis* sp.

### Ethical approval

A written informed consent was obtained from patients prior to the study along with a verbal explanation on the purpose of the study. All experimental protocols and methods were in accordance to the guidelines and regulations of the Medical Ethics Committee of University Malaya Medical Centre, Kuala Lumpur, Malaysia.
